# Elevated resting heart rate as a predictor of inflammation and cardiovascular risk in healthy obese individuals

**DOI:** 10.1038/s41598-021-93449-5

**Published:** 2021-07-06

**Authors:** Fatema Al-Rashed, Sardar Sindhu, Ashraf Al Madhoun, Zunair Ahmad, Dawood AlMekhled, Rafaat Azim, Sarah Al-Kandari, Maziad Al-Abdul Wahid, Fahd Al-Mulla, Rasheed Ahmad

**Affiliations:** 1 Immunology and Microbiology Department, Dasman Diabetes Institute, Al-Soor Street, P.O. Box 1180, 15462 Dasman, Kuwait; 2 Animal and Imaging Core Facility, Dasman Diabetes Institute, Dasman, Kuwait; 3 Royal College of Surgeons in Ireland, Busaiteen, Bahrain; 4 School of Biomedical Sciences, Monash University, Melbourne, Australia; 5 Royal College of Surgeons in Ireland, Dublin, Ireland; 6 Genetics and Bioinformatics Department, Dasman Diabetes Institute, Dasman, Kuwait

**Keywords:** Immunology, Inflammation

## Abstract

The role of leukocyte inflammatory markers and toll like receptors (TLRs)2/4 in pathologies associated with elevated resting heart rate (RHR) levels in healthy obese (HO) individuals is not well elucidated. Herein, we investigated the relationship of RHR with expression of leukocyte-inflammatory markers and TLRs in HO individuals. 58-obese and 57-lean participants with no history of a major medical condition, were recruited in this study. In HO individuals, the elevated-RHR correlated positively with diastolic blood pressure, cholesterol, pro-inflammatory monocytes CD11b^+^CD11c^+^CD206^−^ phenotype (r = 0.52, *P* = 0.0003) as well as with activated T cells CD8^+^HLA-DR^+^ phenotype (r = 0.27, *P* = 0.039). No association was found between RHR and the percentage of CD16^+^CD11b^+^ neutrophils. Interestingly, elevated RHR positively correlated with cells expressing TLR4 and TLR2 (CD14^+^TLR4^+^, r = 0.51, *P* ≤ 0.0001; and CD14^+^TLR2^+^, r = 0.42*, P* = 0.001). TLR4^+^ expressing cells also associated positively with the plasma concentrations of proinflammatory or vascular permeability/matrix modulatory markers including TNF-α (r = 0.36, *P* = 0.005), VEGF (r = 0.47, P = 0.0002), and MMP-9 (r = 0.53, *P* ≤ 0.0001). Multiple regression revealed that RHR is independently associated with CD14^+^TLR4^+^ monocytes and VEGF. We conclude that in HO individuals, increased CD14^+^TLR4^+^ monocytes and circulatory VEGF levels associated independently with RHR, implying that RHR monitoring could be used as a non-invasive clinical indicator to identify healthy obese individuals at an increased risk of developing inflammation and cardiovascular disease.

## Introduction

Obesity is characterized by a state of low-grade chronic inflammation called metabolic inflammation^1^, which contributes to many inflammatory diseases, such as diabetes mellitus, cardiovascular disease^2^, and some types of cancers^3^. Heart rate is controlled via the autonomic nervous system, and a higher resting heart rate (RHR) is explained by impaired autonomic nerve function^4^. The pathophysiology of chronic low-grade inflammation in obesity setting is associated with perturbation in the expression of leukocyte inflammatory markers and toll-like receptors (TLRs) 2/4 along with increased levels of MMP9, VEGF and cytokines such as tumor necrosis factor alpha (TNFα) and interleukin-6 (IL-6)^5–7^. Besides, the autonomic nervous system can be impacted by elevated levels of inflammatory cytokines, such as IL-6 and TNF-alpha^8^. In obesity, an overall increase in the RHR compared to eutrophic group indicates that there is an association between obesity and an elevated RHR^9^. Elevated RHR levels cause micro-inflammation which is involved in the pathogenesis for endothelial dysfunction and cardiovascular pathologies^10^. On the other hand, lower heart rates may benefit conditions, such as congestive heart failure, myocardial infarction, atrial fibrillation, obesity, hyperinsulinemia, insulin resistance, and atherosclerosis. Given these studies, it may be speculated that obese individuals are at a risk of RHR modulation from obesity-associated changes in the autonomic nerve function.

The role of metabolic inflammation in pathologies associated with elevated RHR levels in healthy obese individuals is still not well elucidated. Identifying inflammatory cell populations and related proteins in the circulation can help to clarify the underlying mechanisms associated with elevated RHR levels in such pathologies and thus lead to new therapeutic targets and strategies. Because changes in the expression of inflammatory leukocyte markers including TLRs were observed in obesity, we investigated the relationship of RHR with the expression of the leukocyte inflammatory markers and toll-like receptors (TLRs) 2/4 in healthy obese individuals. Our multiple regression analysis showed that RHR is independently associated with CD14^+^TLR4^+^ monocytes and VEGF expression.

## Materials and methods

### Study population, anthropometry, and data stratification

The study cohort comprised of 58 healthy obese (30 male and 28 female) volunteers with no major medical conditions, alcohol consumption, and smoking. In this study, a lean group comprising of 57 individuals was included only for basic comparison of physical characteristics of the actual study population (healthy obese group).The body mass index (BMI) was measured using the standard formula: BMI = body weight (kg)/height^2^ (m^2^). Written informed consent was obtained from all study participants following ethical guidelines of the Declaration of Helsinki and approval by Kuwait Ministry of Health Ethical Board (2017/542). All participants were given a health questionnaire. The inclusion criteria aimed to involve volunteers without diabetes, chronic or inflammatory diseases, and medication or vitamin consumption. The exclusion criteria consisted of active pregnancy, antihistamine or anti-inflammatory therapy 30 days prior to the study onset, and the use of tobacco, cannabis, recreational drugs, or alcohol a year prior to the study.

Heights and weights were measured using calibrated, portable electronic weighing scales and portable inflexible height-measuring bars and waist circumference was measured using constant-tension tape. Whole-body composition including percent body fat, soft lean mass, and total body water were measured using an IOI 353 Body Composition Analyzer (Jawon Medical, South Korea). ACTi graphical activity (data not shown) was recorded using electronic tri-axial monitor (wGT3X-BT ActiGraph LLC, Pensacola, FL, USA) for seven consecutive days and the RHR was recorded at the baseline. Anthropometric and clinical characteristics of the study participants are summarized in Table 1.

**Table 1:** Physical characteristics of the study population.

Physical characteristics of subjects	Lean group (n = 57)	Obese group (n = 58)	P value
Age (years)	32 ± 5.4	34.6 ± 7.0	0.097
Weight (kg)	63.3 ± 10.5	88.0 ± 13.64	**0.048**
Height (cm)	169.9 ± 11.7	168.2 ± 8.3	0.1545
BMI (kg/m^2^)	21.8 ± 1.6	32.7 ± 5.7	**< 0.0001**
Waist to hip ratio	0.77 ± 0.039	0.84 ± 0.17	**0.0465**
Fat %	23.3 ± 7.1	35.1 ± 5.7	**< 0.0001**
BP/ systolic (mmHg)	105 ± 9.2	113.3 ± 11.6	**< 0.0001**
BP/diastolic (mmHg)	64.9 ± 8.1	70.23 ± 7.9	**< 0.0001**
RHR	66.9 ± 8.1	84.6 ± 4.2	**< 0.0001**

Bold values denote statistical significance.

All values are means ± standard deviations unless labeled otherwise.

*BMI* body mass index, *BP* blood pressure, *RHR* resting heart rate.

Since no significant differences between genders were observed with regard to common risk factors including age, BMI, hip circumference, fat weight, heart rate, as well as fasting glucose and insulin concentrations, triglycerides, total cholesterol, high-density lipoprotein (HDL) cholesterol, and Homeostatic model assessment for insulin resistance (HOMA-IR) (Supplementary Table S1), the data were pooled for further analysis. Based on RHR, the data were stratified into four group quintiles as follows: Q1: 36–64 beats per minute (bpm); Q2: 65–70 bpm; Q3: 71–78 bpm; and Q4: 79–129 bpm.

### Measurement of metabolic and inflammatory markers

Participants were asked to fast for at least 10 h before visit. Blood pressure (BP) and RHR average measurements were taken from three readings taken 1 min intervals apart and after subjects were allowed to rest for at least 10 min. Blood samples were collected in EDTA vacutainer tubes (10 mL, BD Vacutainer system, Plymouth, UK) as described previously. Plasma was separated by centrifugation (400*g* for 5 min), aliquoted and frozen immediately at − 80 °C for further analysis^11,12^.

Blood samples were analyzed for fasting blood glucose (FBG), lipid profile, glycated hemoglobin (HbA1c), and fasting insulin. FBG and lipid profiles (plasma triglycerides, HDL, and cholesterol) were measured using Siemens Dimension RXL chemistry analyzer (diamond Diagnostics Holliston, MA, USA). HOMA-IR, as a homeostatic model assessment of insulin resistance, was calculated from basal (fasting) glucose and insulin concentrations using the following formula: HOMA-IR = fasting insulin (μU/L) × fasting glucose (nmol/L) / 22.5. All assays were performed following the manufacturer’s instructions.

### Enzyme-linked immunosorbent assay (ELISA)

Commercial ELISA kits were used for measuring insulin concentration, C-peptide, (Mercodia, Uppsala, Sweden) and inflammatory cytokines including IL-1β, TNF-α, IL-17A, MCP-1, and VEGF (R&D Systems, Minneapolis, USA) following the manufacturers’ instructions. Quality control sera were used to measure accuracy and precision of the assays.

### Flow cytometry

To determine expression of different leukocyte subsets in the whole blood, multi-color fluorescence-activated cell sorting (FACS) analysis was conducted using freshly collected whole blood samples. Briefly, 1 ml of lysing buffer was added to 0.1 ml of blood sample to eliminate erythrocytes from the remaining peripheral blood leukocyte populations. Cells were washed twice with 1 ml PBS and then incubated with fluorochrome-conjugated mouse anti-human monoclonal antibodies against CD11c, CD11b, CD206, CD8, CD4, HLA-DR, CD16, TLR4, TLR2, and isotype-specific respective control antibodies. Detail of all antibodies with clones is described in Supplementary Table S2). The three gating strategies used to identify and quantify the three distinct leukocyte populations namely neutrophils, monocytes, and lymphocytes are summarized in Supplementary Figure S1. TLR2/4 expression was evaluated on CD14^+^ cells. Data were collected using a BD FACSCanto II flow cytometer and analyzed using DIVA software (version V6.1.3, BD Pharmingen)^12^.

### Statistical analysis

Statistical analysis was performed using GraphPad Prism software (version 6.05; San Diego, CA, USA) and SPSS for Windows version 19.01 (IBM SPSS Inc., USA). The recorded baseline resting heart rate of all participants were divided into quartiles using a similar model presented by Park et al.^13^. Unless otherwise indicated, data are shown as mean ± standard deviations (SD) values. The two groups were compared using *t*-tests for continuous data, and one-way ANOVA—followed by Tukey's test was used to compare more than two groups. Correlations and stepwise multiple linear regression analysis were adjusted for the potential confounders and associations were determined between variables. For all analyses, a P-value < 0.05 was considered significant.

### Institutional Review Board statement

The study was conducted according to the guidelines of the Declaration of Helsinki, and approved by the Institutional Review Board (Kuwait Ministry of Health Ethical Board, 2017/542).

## Results

### Demographic and clinical characteristics of study population

The demographic and clinical characteristics of 58 healthy overweight/obese participants and 57 lean controls are summarized in Table 1. Both groups differed significantly regarding weight, BMI, waist to hip ratio, fat percentage, systolic/diastolic BP, and RHR. To further understand the effect of obesity on RHR, we divided the participants into quartiles based on reported RHR^14,15^. As shown in Table 2, the mean age of all participants was 32.1 ± 4.6 years with no significant differences between RHR quartiles. Similarly, no significant differences in body fat composition, waist, and hip circumferences were observed between RHR groups. Height and weight measurements were significantly higher in Q1 group with the lowest baseline RHR as compared to Q4 group (P < 0.048 and P < 0.0001, respectively). On the other hand, BMI values were higher in Q4 group as compared to Q1 group (P = 0.03); whereas, no differences were observed between groups regarding waist-to-height and waist-to-hip ratios.

**Table 2:** Demographic and clinical characteristics of study population.

Physical characteristics of the subjects	Baseline resting heart rate (quartiles)				*P *value
	1 (36–64)	2 (65–70)	3 (71–78)	4 (79–129)	
Age (years)	29.5 ± 3.11	32 ± 3.33	34.6 ± 7.0	32.52 ± 5.08	0.097
Weight (kg)	97.7 ± 11.9	89.6 ± 17.77	88.0 ± 13.64	82.2 ± 18.0	**0.048**
Height (cm)	178.9 ± 9.2	168.2 ± 12.6	166.9 ± 10.1	160.6 ± 8.3	**≤ 0.0001**
BMI (kg/m^2^)	30.4 ± 2.74	31.7 ± 4.37	31.5 ± 4.0	32.6 ± 5.7	**0.030**
Waist circumference (inch)	35.8 ± 3.34	35.9 ± 6.05	37.9 ± 6.3	37.8 ± 6.1	0.576
Hip circumference (inch)	42.4 ± 3.72	42.5 ± 4.87	43.2 ± 2.6	43.9 ± 5.4	0.0720
Waist to hip ratio	0.84 ± 0.081	0.83 ± 0.10	0.88 ± 0.16	0.86 ± 0.09	0.7849
Waist to hight ratio	0.20 ± 0.02	0.21 ± 0.02	0.22 ± 0.048	0.23 ± 0.03	0.3571
Fat %	33.6 ± 3.92	31.6 ± 2.5	31.1 ± 5.02	31.7 ± 3.06	0.797
BP/systolic (mmHg)	106 ± 9.53	111.7 ± 13.9	112.7 ± 9.5	108.6 ± 6.9	0.983
BP/diastolic (mmHg)	58.0 ± 7.8	64.9 ± 6.85	70.36 ± 9.38	70.6 ± 8.13	**≤ 0.0001**
**Serum metabolic markers**					
Fasting glucose (mmol/l)	4.8 ± 0.46	5.0 ± 0.59	5.56 ± 0.85	5.78 ± 0.585	0.992
Triglycerides (mmol/l)	0.73 ± 0.29	0.88 ± 0.20	1.17 ± 0.58	1.24 ± 0.36	**0.0024**
Total cholesterol (mmol/l)	3.96 ± 0.732	4.95 ± 0.473	4.83 ± 0.977	5.13 ± 0.877	**0.0047**
HDL cholesterol (mmol/l)	1.29 ± 0.265	1.47 ± 0.35	1.37 ± 0.347	1.58 ± 0.344	0.1373
Non-HDL cholesterol (mmol/l)	2.79 ± 0.81	3.76 ± 0.93	3.46 ± 0.96	3.5 ± 1.05	0.1351
Insulin Con. (mu/l)	2.36 ± 1.17	1.70 ± 1.33	1.42 ± 0.72	1.88 ± 1.33	0.2388
HOMA-IR	0.52 ± 0.24	0.36 ± 0.314	0.353 ± 0.23	0.47 ± 0.29	0.3156

Bold values denote statistical significance.

All values are means ± standard deviations unless labeled otherwise.

*BMI* body mass index, *BP* blood pressure, *HR* heart rate, *HDL* high-density lipoprotein, *HOMA-IR* homeostatic model assessment for insulin resistance.

The Q4 group, comprising of individuals with the highest baseline RHR, had significantly elevated levels of triglycerides (P = 0.0024) and total cholesterol (P = 0.0047). While the arterial blood systolic pressure values were comparable in all tested groups, the diastolic BP measurements were notably higher in Q3 and Q4 groups with increased RHR levels (P ≤ 0.0001). The systolic BP differed non-significantly among all groups.

As indicated by analysis of FBG and HOMA-IR values, our study population is non-diabetic. However, those with higher RHR (Q3 and Q4 groups) showed a slight rise in FBG, suggesting a prospective prediabetic status in accordance with the American Diabetes Association (ADA) diagnostic scale; yet, insulin resistance index and HOMA-IR values were within the normal range (less than 1), with no significant differences between groups.

Further multiple regression analysis unveiled that diastolic BP and total cholesterol, but not BMI, triglycerides or insulin were independently associated with RHR (Table 3).

**Table 3:** Multiple linear regression analysis: RHR as dependent variable, relative to clinical parameters.

	Metabolic parameters	Standardized coefficient β	95% confidence interval	*P *value
Resting heart rate (bpm)	BMI (kg/m^2^)	0.3212	− 0.3547 to 0.9593	0.3545
	BP/diastolic (mmHg)	0.1885	0.1192 to 0.8901	**0.0121**
	Triglycerides (mmol/l)	4.175	− 13.73 to 3.348	0.2237
	Total cholesterol (mmol/l)	2.019	0.5186 to 8.779	**0.0287**
	Insulin Con. (mu/l)	0.6633	− 2.633 to 0.08016	0.0642

All values are means ± standard deviations unless labeled otherwise.

*BMI* body mass index, *BP* blood pressure.

Bold values denote statistical significance.

### Association between RHR and leukocyte phenotypes in the peripheral blood

**Figure 1:**
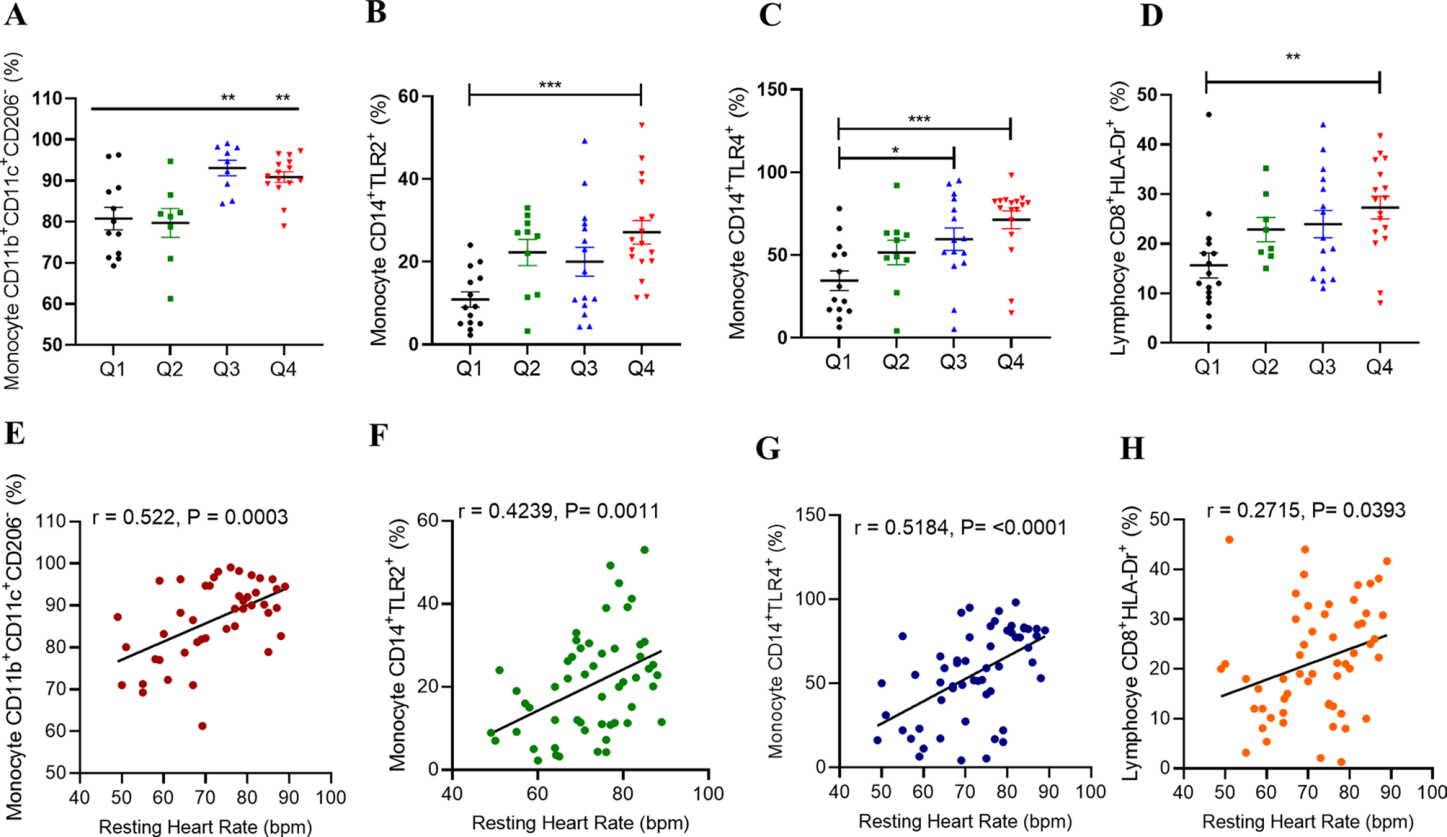
Association between RHR and leukocyte phenotypes in peripheral blood. Flow cytometry analysis was conducted to identify the expression levels of different inflammatory related leukocyte subsets expression cross RHR quartiles. Pro-inflammatory monocytes (**A**), TLR2^+^ monocytes (**B**), TLR4^+^ monocytes (**C**) and cytotoxic T cells (**D**). Pearson's correlation coefficient analysis was conducted to investigate the relationship between RHR and measured leukocyte subsets expression (**E**–**H**). All data are expressed as mean ± SD. P ≤ 0.05 was considered statistically significant (*P < 0.01; **P < 0.001, ***P < 0.0001).

Heart rate variability and leukocytes are associated in the context of metabolic diseases^16,17^. However, the relationship between RHR and different leukocyte subgroups in obesity is still unclear. Herein, we investigated the relationship between leukocyte phenotypes and RHR. Pro-inflammatory monocytes with phenotype CD11b^+^CD11c^+^CD206^−^^18,19^ were found to be significantly elevated in individuals with higher RHR quartiles (Groups Q3 and Q4) (P ≤ 0.01) as compared to lower RHR groups Q1 and Q2 (Fig. 1A).

TLR2/TLR4 and CD14 play a critical role in the innate immune responses^20,21^. Upon activation, these cells secrete pro-inflammatory cytokines and mediate macrophage activation and regulate functioning^20^. To determine whether the RHR levels were associated with changes in monocytic profile, TLR2/TLR4 expression on CD14^+^ cells was evaluated. Interestingly, RHR and percentages of CD14^+^TLR2^+^ and CD14^+^TLR4^+^ monocytes were simultaneously augmented (Fig. 1B,C, respectively). Relative to the Q1 group, Q4 individuals showed significantly higher CD14^+^TLR2^+^ monocyte counts (P = 0.0008), whereas CD14^+^TLR4^+^ monocytes were found to be elevated in both groups Q3 (P = 0.03) and Q4 (P = 0.0004).

Lymphocytic T‐cell‐mediated immunity has been linked to a variety of heart conditions such as myocarditis and post‐myocardial infarction (Dressler's) syndrome^22–24^. Resting heart rate is defined as the number of beats per minute when the individual is at complete rest. It indicates the basic fitness level, therefore, the more well-conditioned the body, the less effort and fewer beats per minute it takes the heart to pump blood to the body at rest. To understand if RHR was related to T cell activation and the expression of cytotoxic T cells, we determined the expression of HLA-DR as a marker of cardiac damage on CD8^+^ T lymphocytes^25^. In this regard, participants with elevated RHR in group Q4 had significantly increased percentages of activated CD8^+^ T cells in the circulation compared to those in group Q1 (P = 0.0064) (Fig. 1D).

However, when we investigated the effect of RHR on mature neutrophils CD16^+^CD11b^+^^26^, there was no significant difference across all four RHR quartiles (Supplementary Figure S2A). To further understand those observations, a correlation analysis revealed significant positive associations of RHR with CD11b^+^CD11c^+^CD206^−^ monocytes, CD14^+^TLR2^+^ monocytes, CD14^+^TLR4^+^ monocytes, and CD8^+^HLA^−^DR^+^ lymphocyte subsets in the circulation (Fig. 1E–H, respectively).

RHR did not associate with CD11b^+^CD16^+^ circulating neutrophils (selective gating is shown in Supplementary Figure S1B). To analyze independent associations of these subsets with RHR, multiple regression analysis was carried out which identified only monocytes as the independent predictors of RHR (Table 4).

**Table 4:** Multiple linear regression analysis: RHR as a dependent variable, relative to monocyte surface markers.

	Leukocyte surface expression	Standardized coefficient β	95% confidence interval	*P *value
Resting heart rate (bpm)	Monocyte CD11b^+^CD11c^+^CD206^−^ (%)	0.4875	0.1739–0.8010	**0.0032**
	Lymphocyte CD8^+^HLA-DR^+^ (%)	0.1527	− 0.09623–0.4017	0.2217
	Monocyte CD14^+^TLR4^+^ (%)	0.1526	0.04385–0.2613	**0.0072**
	Monocyte CD14^+^TLR2^+^ (%)	0.2297	0.02261–0.4368	**0.0307**

Bold values denote statistical significance.

### Association between RHR and inflammatory markers

**Figure 2:**
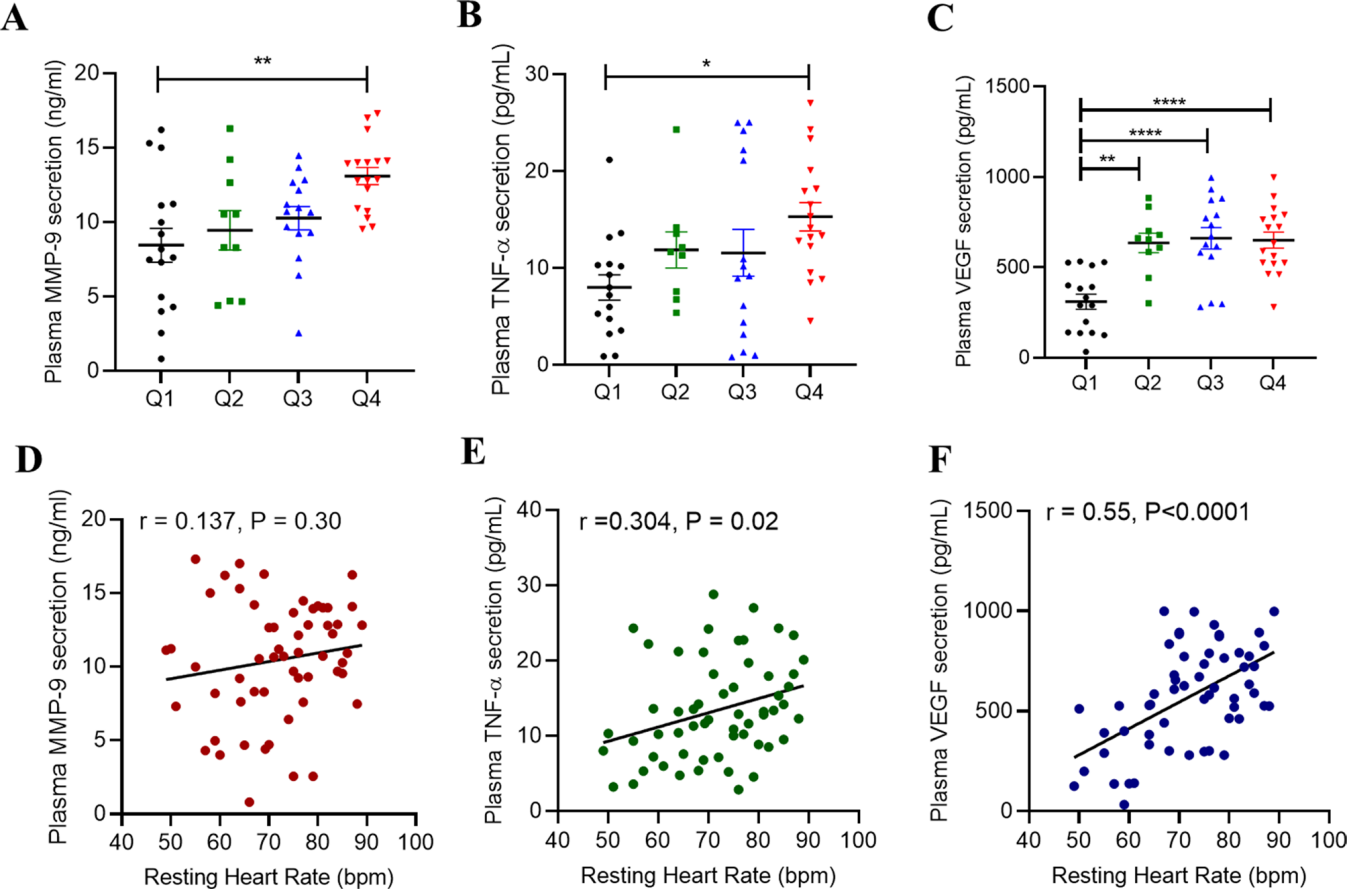
Elevated RHR is associated with obesity-inflammatory markers in the plasma. Plasma levels of obesity related cytokines secretion of MMP-9 (**A**), TNF-α (**B**) and VEGF (**C**) was determined by ELISA. Pearson's correlation coefficient and linear regression analysis was conducted to investigate the relationship between secreted cytokines and RHR (**D**–**F**). All data are expressed as mean ± SD. P ≤ 0.05 was considered statistically significant (*P < 0.01; **P < 0.001, ***P < 0.0001).

Increased plasma levels of pro-inflammatory cytokines in obese individuals were found to be associated with cardiac disorders^27–29^. Therefore, we next investigated the correlation between levels of critically important cytokines and the RHR. Group Q4 with the highest RHR was found to have significantly elevated plasma levels of MMP-9 (P = 0.006) and TNF-α (P = 0.042) compared to group Q1 with the lowest RHR, as shown in Fig. 2A,B, respectively. A gradual surge was also noted in the levels of plasma VEGF corresponding to an increase in the RHR (Fig. 2C). RHR associated with the plasma levels of VEGF (r = 0.55, P < 0.0001) and TNF-α (r = 0.304, P = 0.02), but not with plasma MMP-9 (r = 0.137, P = 0.30) (Fig. 2D–F). As indicated by multi-linear regression analyses, only plasma VEGF levels associated independently with RHR (Table 5). However, plasma levels of IL-1β and IL-17 were comparable among groups and those of IL-6 and IL-8 were found to be elevated in higher RHR quartile groups Q3 and Q4, albeit the differences were non-significant (Supplementary Table S3).

**Table 5:** Multiple linear regression analysis: RHR as a dependent variable, relative to serum inflammatory factors.

	Inflammatory plasma secretions	Standardized coefficient β	95% confidence interval	*P *value
Resting heart rate (bpm)	MMP9 (ng/ml)	0.03590	− 0.6426 to 0.7144	0.9159
	VEGF (pg/ml)	0.02184	0.01113 to 0.03255	**0.0001**
	TNF-a (pg/ml)	0.07964	− 0.3416 to 0.5009	0.7060

Bold values denote statistical significance.

### Relationship between plasma VEGF levels and monocyte immunophenotypes

**Figure 3:**
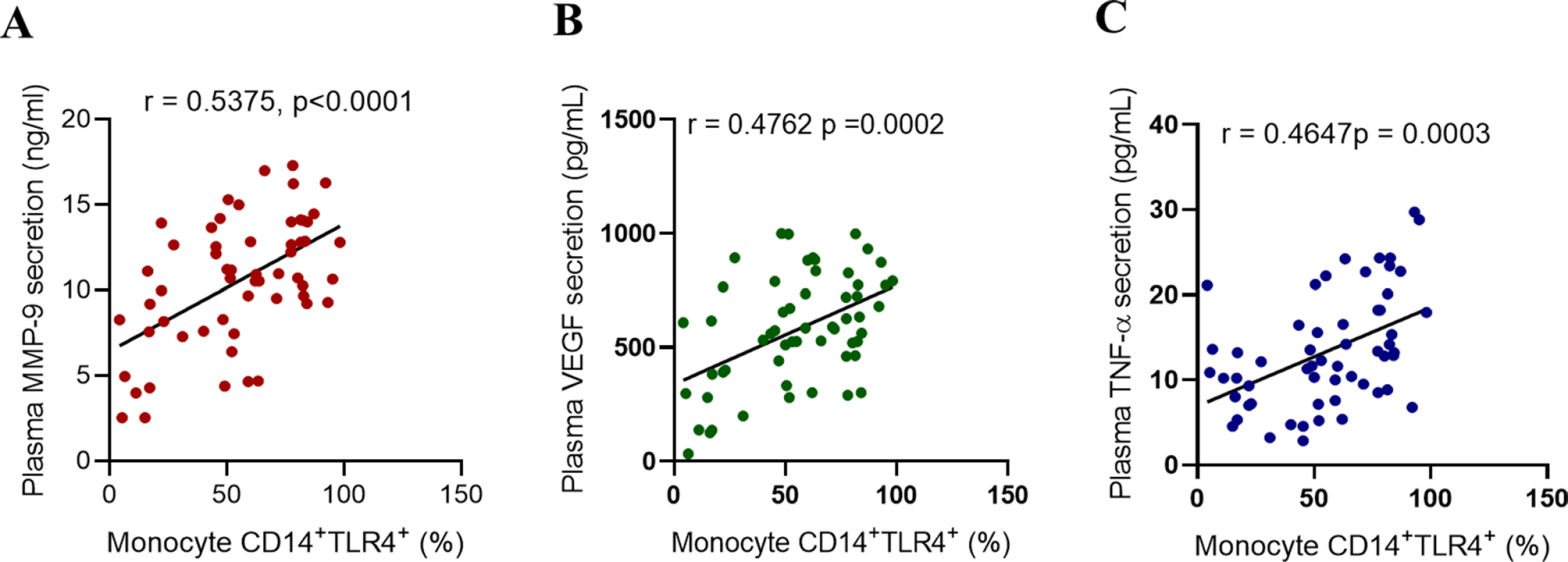
CD14^+^TLR4^+^ monocytes are associated with obesity related-inflammatory markers in the plasma. Pearson's correlation coefficient analysis was conducted to investigate the association between monocyte TLR4 expression and plasma levels of obesity related cytokines secretion of MMP-9 (**A**), VEGF (**B**) and TNF-α (**C**). All data are expressed as mean ± SD. P ≤ 0.05 was considered statistically significant (*P < 0.01; **P < 0.001, ***P < 0.0001).

Given that plasma VEGF level was a strong predictor of RHR, we next conducted multiple linear regression analysis to find which monocyte phenotypes(s) were associated independently with plasma VEGF levels in the circulation. As shown in Table 6, among the three monocyte phenotypes, the CD14^+^TLR4^+^ subset was the only cell type that associated independently with plasma VEGF levels. As expected, percentages of CD14^+^TLR4^+^ monocytes associated with plasma levels of MMP-9 (r = 0.538, P < 0.0001), VEGF (r = 0.476, P = 0.0002), and TNF-α (r = 0.465, P = 0.0003) (Fig. 3).

**Table 6:** Multiple linear regression analysis: VEGF as a dependent variable, relative to monocyte surface markers.

Leukocyte surface expression	Plasma VEGF secretion (pg/ml)		
	Standardized coefficient β	95% confidence interval	P value
Monocyte CD11b^+^CD11c^+^CD206^−^ (%)	− 3.223	− 11.74 to 5.298	0.4486
Monocyte CD14^+^TLR4^+^ (%)	4.877	1.880 to 7.874	0.0021*
Monocyte CD14^+^TLR2^+^ (%)	4.789	− 0.9174 to 10.49	0.0975

All values are means ± standard deviations unless labeled otherwise.

*VEGF* vascular endothelial growth factor.

**Figure 4:**
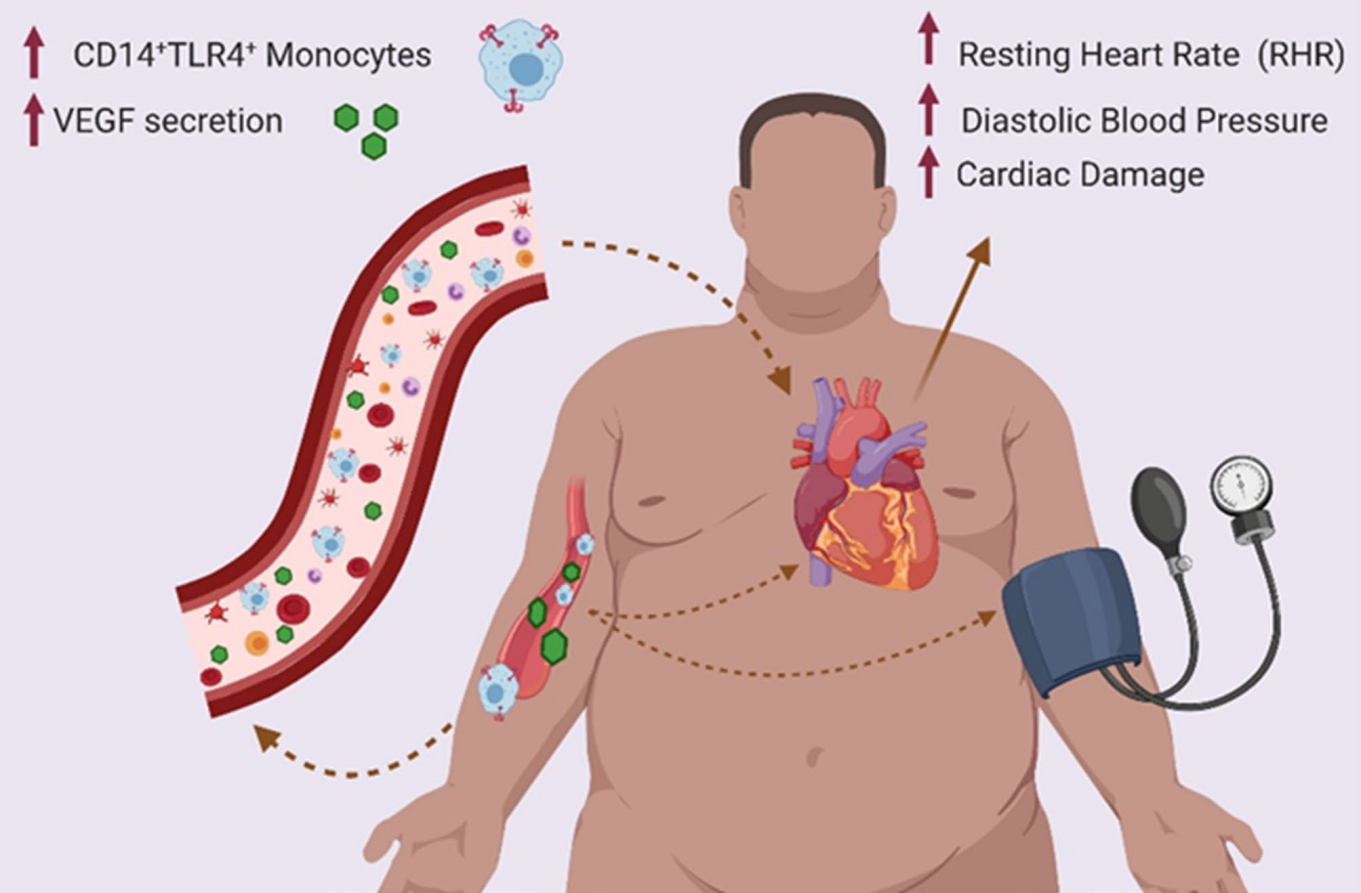
Schematic illustration. Elevated RHR in healthy obese individuals is associated with CD14^+^TLR4^+^ monocytes and circulatory VEGF levels. Tracking and monitoring changes in the RHR could be used as a useful and non-invasive clinical indicator to identify the obese individuals at risk of sub-clinical inflammation and ensuing cardiovascular events. The figure was created using BioRender.com.

Overall, our results show that in healthy obese individuals, increased CD14^+^TLR4^+^ monocytes and circulatory VEGF levels associated independently with RHR, suggesting that RHR monitoring could be used as a non-invasive clinical indicator to identify healthy obese individuals at risk for inflammation and cardiovascular disease (Fig. 4).

## Discussion

In this study we report, for the first time to our knowledge, an association between RHR and immunological changes in healthy middle-aged obese individuals. Heart rate denotes the cardiac modulation by sympathetic and parasympathetic (vagal) components of the autonomic nervous system. Heart rate levels have been associated with general wellbeing and mortality^30^; while, the elevated heart rate is considered a potential risk marker of cardiovascular disease^31^. RHR was reported to be a long-term risk factor of all-cause mortality in hypertensive patients^32^ as well as in healthy populations^33^; and it has been extensively studied in the context of diabetes^34,35^, hypertension and heart failure^36,37^, and different physical exercise training and fitness programs and their duration^38,39^. Obesity is a critical risk factor of diabetes, independent of other risk factors such as hypertension, hyperlipidemia, and related conventional risk factors. During obesity, upregulation of chronic inflammatory responses has been documented^40,41^; and the onset of obesity has been linked to elevated RHR when compared with normal weight individuals^42^. Excessive consumption of high calorie diets generates the oxidative stress, leading to inflammation, sympathoexcitation, and RHR elevation. RHR both reflects (risk marker) and contributes to (risk factor) cardiac pathology^43^. In our study population, a comparable body fat ratio was observed across all RHR groups; however, the increase in BMI from Q1 to Q4 was moderately significant. Since BMI has been reported to overestimate obesity, we also included waist-to-height and waist-to-hip ratios as indicators of obesity^44^. However, waist-to-height and waist-to-hip ratios showed no significant differences across Q1 and Q4 RHR groups. It implies that the obesity per se might have had an indirect impact in modulating RHR in our study population as RHR quartiles differed non-significantly with respect to waist and hip circumferences, waist to hip ratio, and body fat percentage. A 10-year follow-up study by Zhang et al. investigating the cumulative effect of overweight and RHR on the incidence of pre-diabetes and diabetes in 1729 participants reported that being overweight was an independent risk factor of prediabetes and diabetes, whereas a status of being overweight with a faster RHR further increased the risk of prediabetes and diabetes^45^.

The effect of RHR on BP and metabolic syndrome (MetS) risk factors has been studied previously^14^. In our study population, both systolic and diastolic BPs were within the healthy ranges. Although, the systolic BP varied non-significantly across RHR quartiles, the diastolic BP was significantly elevated in Q4 group with the highest RHR compared to Q1 group with the lowest RHR (P < 0.0001). Likewise, all our study participants were within the normal range regarding levels of blood triglyceride and total cholesterol, both indices differed significantly between quartiles Q4 and Q1. As per multivariate analysis, diastolic BP, triglycerides and total cholesterol independently predicted RHR in our study population. In agreement with our data showing a congruence between RHR and diastolic BP, a large study by Liu et al., comprising of 8000 individuals, found that high RHR associated with high BP values^46^. Similarly, corroborating our results, Kwok et al.^47^ and Christofaro et al.^48^ also reported a direct association between elevated RHR and increased BP in the young individuals. In agreement, at least in part, with our data showing an association between RHR and levels of triglycerides and total cholesterol, Williams et al. found that RHR associated positively with plasma concentrations of triglycerides, VLDL cholesterol, and VLDL mass^49^. Likewise, another large-scale study of 11,876 male adults found that elevated RHR was positively associated with triglycerides, systolic/diastolic blood pressure, BMI, and fasting blood glucose levels, concluding that the relative risk of metabolic syndrome steadily increased as the RHR increased^14^. In contrast to this study, however, we did not find a positive association of RHR with BMI and fasting blood glucose and this discrepancy may be due to cohort differences between two studies with regard to age and adiposity.

Given that RHR was found to be associated with chronic low-grade inflammation and microinflammatory responses in apparently healthy men as well as in those with atherothrombotic risk factors^50^, we aimed to understand the prognostic significance of RHR as a marker and co-variate of metabolic inflammation. In our healthy obese study cohort, we found that in absence of a targeted physical activity or medications, the individuals with elevated RHR (quartiles Q3 and Q4) had concomitantly increased numbers of proinflammatory CD11b^+^CD11c^+^CD206^−^ and CD14^+^TLR4^+^ monocyte subsets in the circulation. Similarly, the CD14^+^TLR2^+^ monocyte subset was also found to be significantly increased in individuals with high RHR (quartiles Q2 and Q4) compared to low RHR individuals (quartile Q1). Of note, TLR2/4 activation and signaling have been implicated with inflammation^51^, insulin resistance^52^, developmental and adult neuroplasticity^53^, ischemic injury to heart and brain^54^ as well as thermoregulation and fever after infections^55^. Our data showing a positive association between increased numbers of TLR2/4-bearing monocytes in the individuals with an elevated RHR point to a plausible link between TLRs and the regulation of sympathetic/parasympathetic components of the autonomic nervous system. Consistent with this notion, Okun et al. showed that the mice lacking TLR2 or TLR4 had lower basal HR due to the increased parasympathetic tone with changes in body temperature and energy metabolism^56^. In further deciphering the impact of cellular changes in these individuals, we measured inflammatory cytokines and found that TNF-α, MMP-9, and VEGF levels were significantly elevated in individuals with higher RHR (quartile Q4) as compared to those with lower RHR (quartile Q1); however, only the VEGF levels independently predicted the RHR. Indeed, increased levels of TNF-α, IL-6, MMP-9, and VEGF have been extensively documented in obesity and found to be associated with metabolic inflammation, hyperglycemia, insulin resistance and progression to type-2 diabetes or metabolic syndrome^7,57–61^. Moreover, consistent with our observations at least in part, circulatory monocytes in obesity have been shown to overexpress the M1 pro-inflammatory CD11c and infiltration CD11b markers, which associated with plasma levels of pro-inflammatory cytokines in both mice and humans^62,63^. In our study cohort, circulatory levels of other proinflammatory cytokines/chemokines including IL-1β, IL-6, IL-8, IL-17A, and MCP-1 did not differ significantly between high RHR (quartile Q4) and low RHR (quartile Q1) groups which may be due to the reason that these individuals were a healthy obese population and, therefore, the majority of inflammatory cytokines were found to be unaffected, leading us to speculate that TNF-α, secreted predominantly by activated monocytes/macrophages, could be a more sensitive and relevant marker for monocyte subset changes that were detected in our study cohort. The significant upregulation in the risk factors or markers of cardiovascular damage, such as MMP-9 and VEGF levels, corresponded with the increase in CD14^+^TLR2^+^ and CD14^+^TLR4^+^ monocyte subsets in individuals with high RHR (quartiles Q3 and Q4), suggesting that meta-inflammatory and sympathetic activation changes in these individuals might be intertwined.

TLRs are well-characterized innate immune receptors that are emerging as nutrient sensors and key players in metabolic inflammation. TLR2/4 activation in obesity and associated metabolic syndrome is followed by triggering of dynamic signaling cascades leading to the release of proinflammatory mediators^64^. Of note, the upregulation of TLR4 has been reported to contribute to cardiac failure and was associated with hypertension^65^. Indeed, within our healthy obese population, we found that the circulatory CD14^+^TLR4^+^ monocyte subset independently predicted the VEGF secretion, which is a protein known to accelerate the development of cardiac dysfunction^66^. Furthermore, elevated plasma levels of VEGF are critical in cardiac vascularization and function and its expression is essential for the regulation of coronary microvasculature during changes in oxygen needs^67^. The VEGF expression and secretion in coronary microvascular endothelial cells and cardiomyocytes subjected to stretch was upregulated^68,69^. Notably, VEGF upregulation was also found to be associated with the activation of inflammatory and oxidative stress signaling pathways including TLR4/NF-κB and HIF1α, respectively^70,71^. Together, our data support a risk model for the healthy obese population wherein chronically elevated RHR per se may be a risk factor or early sign of cardiovascular pathologies.

## Conclusions

Our data show that in healthy obese individuals, elevated RHR is independently associated with diastolic blood pressure and total cholesterol levels. RHR also correlated positively with proinflammatory M1 monocytes, activated CD8^+^ T lymphocytes, and peripheral levels of important inflammatory mediators. In our study cohort, increased circulatory VEGF levels predicted the elevated RHR whereas, CD14^+^TLR4^+^ monocyte subset was found to be independently associated with VEGF expression in these individuals. As implied from these data, tracking and monitoring changes in the RHR could be used as a useful and non-invasive clinical indicator to identify the obese individuals at risk of sub-clinical inflammation and ensuing cardiovascular events.

## Supplementary Information

Supplementary Information.
