# Supplementary material for: Elevated resting heart rate as a predictor of inflammation and cardiovascular risk in healthy obese individuals
Source: Sci Rep. 2021 Jul 6;11:13883. doi: 10.1038/s41598-021-93449-5 (PMC8260607; doi:10.1038/s41598-021-93449-5)
Supplement: Supplementary file 1 — Supplementary Information. [file 41598_2021_93449_MOESM1_ESM.pdf]

Title: **Elevated resting heart rate as a predictor of inflammation and cardiovascular risk in healthy obese individuals**

Authors: Fatema Al-Rashed[1], Sardar Sindhu[2], Ashraf Al Madhoun[2], Zunair Ahmad[3], Dawood AlMekhled[4], Rafaat Azim[3], Sarah Al-Kandari[1], Maziad Al-Abdul Wahid[5], Fahd Al-Mulla[6], Rasheed Ahmad[1*]

Affiliations: [1]Immunology & Microbiology Department, Dasman Diabetes Institute, Kuwait; [2] Animal and Imaging Core Facility, Dasman Diabetes Institute, Kuwait. [3]Royal College of Surgeons in Ireland –Bahrain. [4]School of Biomedical Sciences, Monash University, Melbourne, Australia. [5]Royal College of Surgeons in Ireland – Ireland. [6]Genetics and Bioinformatics Department, Dasman Diabetes Institute, Kuwait

Running title: Elevated RHR is an indicator for inflammation/CVD

*Correspondence: Rasheed Ahmad PhD., Immunology & Microbiology Department, Dasman Diabetes Institute, Al-Soor Street, Kuwait, P.O. Box 1180 Dasman, 15462 Kuwait. Phone: 965 2224 2999 Ext. 4311; Fax: 965 2249 2406; E-mail: rasheed.ahmad@dasmaninstitute.org

Supplementary Table S1: Demographic and clinical characteristics of study population, stratified by gender

**Demographic parameters**	Male (n= 30)	Female (n=28)	p-value
Age (years)	33.6 ± 5.65	32.00 ± 3.35	0.7537
Weight (kg)	102.6 ± 9.9	87 ± 10.77	**0.0076****
Height (cm)	174.15 ±8.73	164 ± 9.03	0.0616
BMI (kg/m2)	33.16 ± 2.81	33 ± 3.07	0.3751
Waist circumference (inch)	45.78 ± 5.09	39 ± 4.62	**0.0056****
Hip circumference (inch)	46.94 ± 3.39	47 ± 3.38	0.4921
Fat weight (kg)	34.05 ± 8.14	37 ± 7.1	0.2584
Lean weight (kg)	61.14 ± 7.59	50 ± 8.6	**0.0138***
fat %	31.16 ± 4.42	39 ± 3.9	**0.0429***
BP/ systolic (mmHg)	123.9± 10.84	117 ± 7.8	0.2087
BP/diastolic (mmHg)	75 ± 8.9	73 ± 11.1	0.9923
HR	76.4 ± 8.44	76 ± 6.4	0.6613
**Clinical parameters**			
Fasting glucose (mmol/l)	5.37 ± 0.726	5.0 ± 0.69	0.8725
Triglycerides (mmol/l)	0.93± 0.22	1.0 ± 0.16	0.6151
Total cholesterol (mmol/l)	5.47 ± 1.07	5.0 ± 0.50	0.8359
HDL cholesterol (mmol/l)	1.31 ± 0.240	1.0 ± 0.255	0.9811
Insulin Con. (mu/l)	2.46 ± 1.54	5 ± 1.3	0.1107
HOMA-IR	0.9 ± 0.40	1 ± 0.052	0.0610

All values are means ± standard deviations unless labeled otherwise
BMI: Body mass index; BP: Blood pressure; HR: Heart rate; HDL: High-density lipoprotein; HOMA-IR: Homeostatic model assessment for insulin resistance

Supplementary Table S2: List of Antibodies with detailed information

Antibody	Clone	Host/Isotype	Cat #	Manufacture
**Anti-TLR4-FITC**	HTA125	IgG2a	NB100-56059	Novus Biologicals
**Anti-TLR2-PE**	TL2.1	IgG2a	NBP2-24909	Novus Biologicals
**Anti-CD11c-PE-Cy7**	3.9	Mouse / IgG1, kappa	25-0116-42	eBioscience
**Anti-CD11b-FITC**	Bear1	IgG1 Mouse	IM0530	Beckman Coulter
**Anti-HLA-Dr-PE**	Immu-357	IgG1 Mouse	IM1639	Beckman Coulter
**Anti-CD206-APC**	19.2	Mouse IgG1, κ	550889	BD biosciences/ BD Pharmingen
**Anti-CD14-PE-CF594**	MϕP9	Mouse BALB/c IgG2b, κ	562334	BD biosciences /BD Horizon
**Anti-CD8-Alexa Flour®488**	RPA-T8	Mouse IgG1, κ	557696	BD biosciences/ BD Pharmingen
**Anti-CD4-APC**	RPA-T4	Mouse IgG1, κ	555349	BD biosciences/ BD Pharmingen
**Anti-CD16-APC**	eBioCB16 (CB16)	Mouse / IgG1, kappa	17-0168-42	eBioscience

Supplementary Table S3: Plasma pro-inflammatory cytokines, stratified by RHR

Inflammatory cytokines	Baseline resting heart rate quartile				
	1 (36- 64)	2 (65-70)	3 (71 -78)	4 (79- 129)	P value
Il-1β (pg/ml)	5.0 ± 4.0	4.24 ± 3.25	5.19 ± 9.4	3.96 ± 4.04	0.957
IL-6 (pg/ml)	6.7 ± 7.4	7.24 ± 7.1	10.5 ± 10.48	6.3 ± 8.4	0.655
IL-8 (pg/ml)	5.95 ± 2.9	9.9 ± 8.5	21.5 ± 15.25	14.59 ± 18.9	0.127
MCP-1 (pg/ml)	375.7 ± 167.4	430.3 ± 218.9	543.7 ± 190.2	510 ± 222.0	0.07
VEGF (pg/ml)	310.2 ± 167.0	624.15 ± 6.05	661.3 ± 229.8	650.7 ± 183	**<0.0001******
IL-17A (pg/ml)	33.7 ± 20.9	35.9 ± 25.2	33.9 ± 29.59	33.4 ± 29.3	0.992
MMP-9 (ng/ml)	10.58 ± 4.6	9.46 ± 4.15	10.2 ± 3.0	12.8 ± 2.1	**0.0068****
TNF-α (pg/ml)	10.79 ± 6.6	12.7 ± 6.0	14.3 ± 7.1	15.3 ± 6.0	**0.042***

All values are means ± standard deviations unless labeled otherwise
MCP-1: Monocyte chemoattractant protein-1; VEGF: Vascular endothelial growth factor;
MMP-9: Matrix metalloproteinase-9; TNF-α: Tumor necrosis factor-α

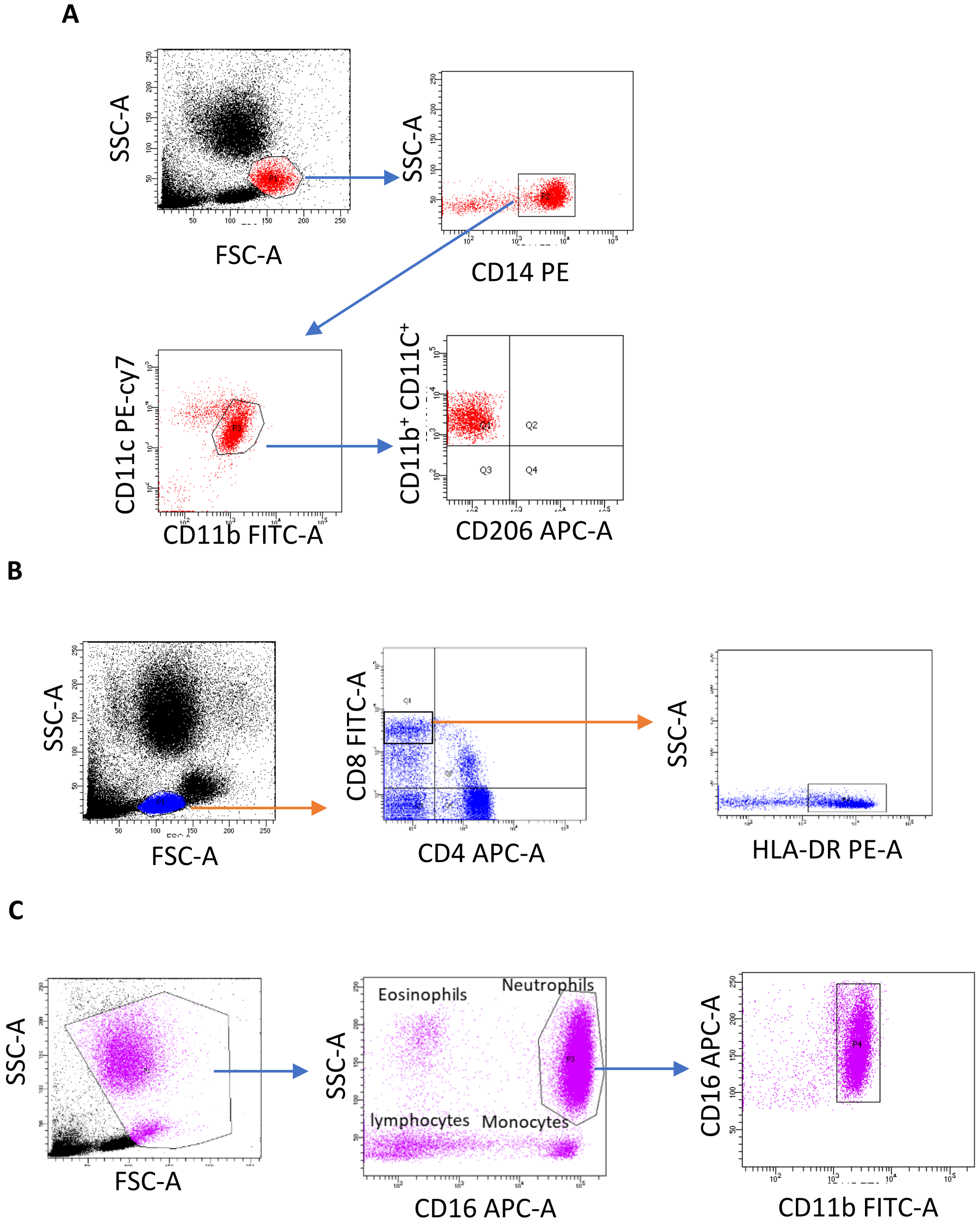

Supplemental Figure. 1. Gating strategy. Cells were gated based on forward and side scatters with negative (dump) selection. From monocytes labeling, subsets were identified based on $CD14^{+}$ expression. (A) The identity of the pro-inflammatory monocyte (red) subsets was validated using surface expression of CD11c, CD11b and negative expression of CD206. To identify the expression of TLR4 and TLR2, similar strategy was used to identify $CD14^{+}TLR4^{+}$ or $CD14^{+}TLR2^{+}$. (B) For total lymphocytes (blue) T cells, cytotoxic T cells were identified based on CD4/CD8 expression. lymphocyte populations were identified based on FSC vs SSC scatter. T-cells were further subdivided in $CD8^{+}$ Tc (cytotoxic T cells) cells and $CD4^{+}$ Th (helper T cells) cells. $CD8^{+}$ Tc were gated for HLA-Dr to identify Cytotoxic T lymphocyte populations. (C) For neutrophil gating (pink), population was identified based in FSC vs SSC scatter followed by further differentiation through their expression of $CD16^{+}$ to remove eosinophils. Mature neutrophils were then identified by their $CD16^{+}CD11b^{+}$ expression.

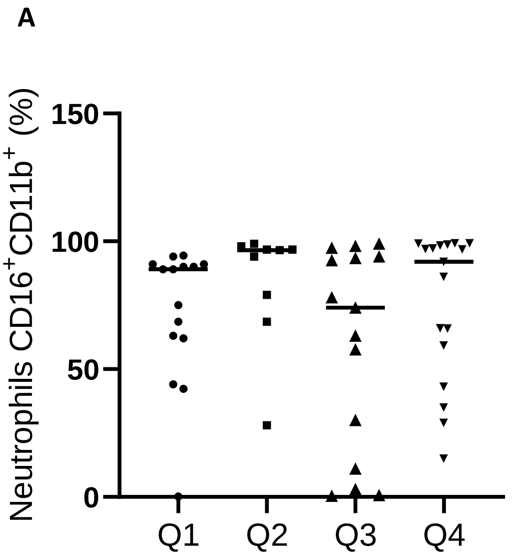

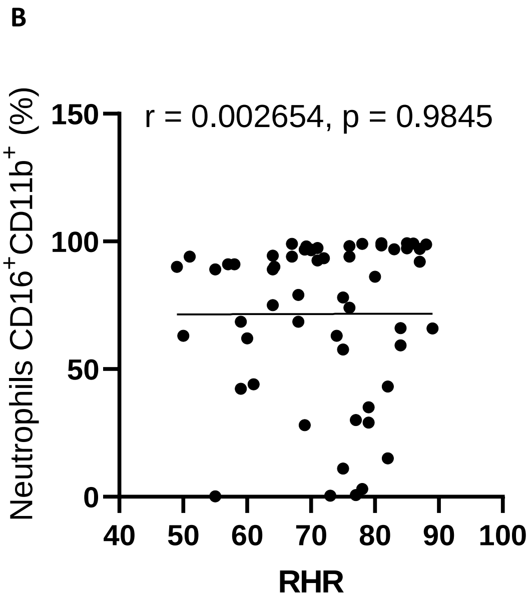

Supplemental Figure. 2 Elevation of RHR have no effect on circulating neutrophils levels. Flow cytometry analysis was conducted to identify the expression levels $CD16^{+}$ $CD11b^{+}$ neutrophils cross RHR quartiles (A). Pearson's correlation coefficient analysis was conducted to investigate the relation between RHR and CD16+ $CD11b^{+}$ (B). All data are expressed as mean ± SD. P≤0.05 was considered statistically significant (*P<0.01; **P< 0.001, ***P< 0.0001).
